# Integrative metabolomics and transcriptomics analysis reveals novel therapeutic vulnerabilities in lung cancer

**DOI:** 10.1002/cam4.4933

**Published:** 2022-06-08

**Authors:** Jose Thaiparambil, Jianrong Dong, Sandra L. Grimm, Dimuthu Perera, Chandra Shekar R. Ambati, Vasanta Putluri, Matthew J. Robertson, Tajhal D. Patel, Brandon Mistretta, Preethi H. Gunaratne, Min P. Kim, Jason T. Yustein, Nagireddy Putluri, Cristian Coarfa, Randa El‐Zein

**Affiliations:** ^1^ Houston Methodist Cancer Center Houston Texas USA; ^2^ Center for Precision and Environmental Health Baylor College of Medicine Houston Texas USA; ^3^ Molecular and Cellular Biology Department Baylor College of Medicine Houston Texas USA; ^4^ Dan L Duncan Comprehensive Cancer Center Baylor College of Medicine Houston Texas USA; ^5^ Advanced Technology Cores Baylor College of Medicine Houston Texas USA; ^6^ Texas Children’s Cancer and Hematology Centers and The Faris D. Virani Ewing Sarcoma Center Baylor College of Medicine Houston Texas USA; ^7^ Department of Biology and Biochemistry University of Houston Houston Texas USA; ^8^ Division of Thoracic Surgery, Department of Surgery Houston Methodist Hospital Houston Texas USA; ^9^ Integrative Molecular and Biological Sciences Program Baylor College of Medicine Houston Texas USA

**Keywords:** adenocarcinoma of lung, carcinoma, non‐small‐cell lung, carcinoma, squamous cell, drug repositioning, lung neoplasms, metabolomics, systems biology, transcriptome

## Abstract

**Background:**

Non‐small cell lung cancer (NSCLC) comprises the majority (~85%) of all lung tumors, with lung adenocarcinoma (LUAD) and squamous cell carcinoma (LUSC) being the most frequently diagnosed histological subtypes. Multi‐modal omics profiling has been carried out in NSCLC, but no studies have yet reported a unique metabolite‐related gene signature and altered metabolic pathways associated with LUAD and LUSC.

**Methods:**

We integrated transcriptomics and metabolomics to analyze 30 human lung tumors and adjacent noncancerous tissues. Differential co‐expression was used to identify modules of metabolites that were altered between normal and tumor.

**Results:**

We identified unique metabolite‐related gene signatures specific for LUAD and LUSC and key pathways aberrantly regulated at both transcriptional and metabolic levels. Differential co‐expression analysis revealed that loss of coherence between metabolites in tumors is a major characteristic in both LUAD and LUSC. We identified one metabolic onco‐module gained in LUAD, characterized by nine metabolites and 57 metabolic genes. Multi‐omics integrative analysis revealed a 28 metabolic gene signature associated with poor survival in LUAD, with six metabolite‐related genes as individual prognostic markers.

**Conclusions:**

We demonstrated the clinical utility of this integrated metabolic gene signature in LUAD by using it to guide repurposing of AZD‐6482, a PI3Kβ inhibitor which significantly inhibited three genes from the 28‐gene signature. Overall, we have integrated metabolomics and transcriptomics analyses to show that LUAD and LUSC have distinct profiles, inferred gene signatures with prognostic value for patient survival, and identified therapeutic targets and repurposed drugs for potential use in NSCLC treatment.

## Graphical abstract

Integrated metabolomic and transcriptomic strategies in lung adenocarcinoma (LUAD) and squamous cell carcinoma (LUSC) tumors. The transcriptomic and metabolomic data were integrated and enriched pathways were inferred. Integrated gene signatures were evaluated for tumor prognosis and drug repurposing.

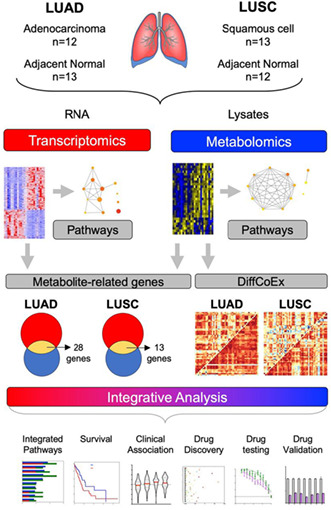

## 1 INTRODUCTION

Non‐small cell lung cancer (NSCLC) is the leading cause of cancer deaths worldwide in both men and women.^1^ NSCLC has several subtypes, with the two major subtypes being adenocarcinoma (LUAD) and squamous cell carcinoma (LUSC), which together account for 85% of primary lung cancers.^2^ LUAD is the most prevalent subtype in non‐smokers,^3^ while LUSC is closely related to smoking.^4^ Historically, NSCLC subtypes were treated similarly due to limited knowledge of the biological differences between LUAD and LUSC, but RNA sequencing (RNA‐Seq) has identified further subtypes within LUAD and LUSC.^5^, ^6^ Major LUSC subtypes identified by The Cancer Genome Atlas (TCGA) agree well with DNA methylation and microRNA profiles,^7^ but the major subtypes of LUAD interact, but do not overlap completely, with epigenetic and microRNA profiles.^8^ Therefore, additional omics profiling technologies would further elucidate the distinct biology of each subtype.

Metabolic alterations are emerging as hallmarks of cancer,^9^, ^10^ including upregulation of the pentose phosphate pathway, fatty acid synthesis pathway, and glutamine maintenance.^11^, ^12^ Lung cancer metabolomic studies were initially focused on differences between cancer patients and healthy individuals using serum and urine metabolites,^13^, ^14^ as well as metabolites from NSCLC tumors.^15^ Integration of metabolomics and differential expression of metabolite‐related genes could provide insight into targeted therapies for personalized medicine. Recent studies in NSCLC have elucidated protein biomarkers,^16^ a six‐gene prognostic signature for disease‐free survival and overall survival,^17^ an RNA metabolic signature for LUAD,^18^ and a prognostic epigenomic NSCLC classifier based on the TCGA methylation spectrum.^19^ However, none of the studies have reported prognostic metabolic signatures or therapeutic targets specifically for LUAD and LUSC.

**FIGURE 1:**
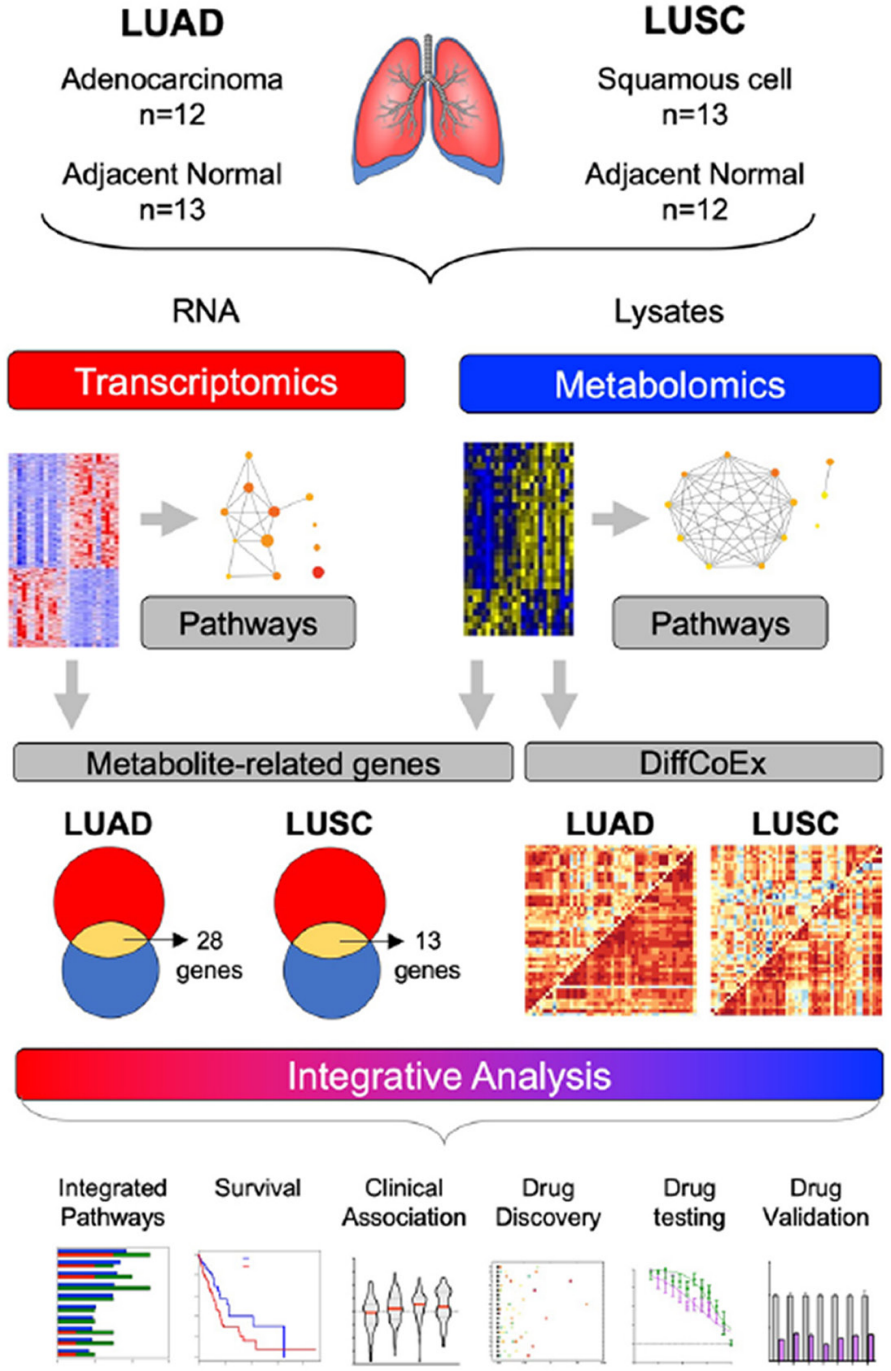
Integrated metabolomic and transcriptomic strategy. We considered biopsies of LUAD tumors (*N* = 13) and adjacent normal tissues (*N* = 12), and LUSC tumors (*N* = 12) and adjacent normal tissues (*N* = 13), and generated RNA‐sequencing and metabolomics profiles. The transcriptomic and metabolomic data were integrated by identifying coordinated changes between tumor and matched normal samples via either differential expression or differential co‐expression, and enriched pathways were inferred. We determined integrated gene signatures, evaluated them for tumor prognosis and drug repurposing, and assessed novel candidates for LUAD treatment. LUAD, lung adenocarcinoma; LUSC, lung squamous cell carcinoma.

In the present study, we aim to define metabolic gene signatures for LUAD and LUSC and identify genes associated with survival, potentially identifying metabolite‐related biomarkers predicting poor outcomes, which might improve treatment for NSCLC. As outlined in Figure 1, by integrating gene expression and metabolomic profiles, we inferred multi‐modal signatures specific for LUAD and LUSC, and further identified metabolic gene survival markers in LUAD and demonstrated their association with tumor stages. Finally, we demonstrated that suppression of therapeutic targets with chemopreventive agents can prioritize the repurposing of existing anti‐cancer drugs, as evidenced by the anti‐cancer effects of the drug AZD‐6482 in LUAD.

## 2 MATERIALS AND METHODS

### 2.1 Biological samples

LUAD and LUSC tissue samples (15 each) for both with adjacent normal tissues (60) were obtained from the Houston Methodist biorepository under an IRB‐approved protocol. All patients signed an informed consent for the use of their tissues in research studies.

### 2.2 RNA sequencing

After measuring concentrations using Thermo Scientific's Nanodrop, RNA was sent to the Sequencing and Gene Editing Core at the University of Houston for: RNA quality analysis using the Agilent 2100 Bioanalyzer; sequencing library preparation using the Illumina Truseq stranded total RNA kit, with 1000 ng of RNA per sample with a RIN value >8.4; and sequencing using the NextSeq 500 (Illumina).

### 2.3 Transcriptomics data analysis

RNA‐seq data were mapped onto the human genome UCSC hg19 using hisat2 (HISAT2, RRID:SCR_015530),^20^ and gene expression were quantified using StringTie (StringTie, RRID:SCR_016323) and GENCODE model. Differentially expressed genes were detected using unequal variance two‐sided Student's *t*‐test with the Benjamini‐Hochberg correction, with significance achieved at FDR < 0.05 and fold change exceeding 1.25X. Enriched pathways were inferred using the hypergeometric test, with significance achieved for FDR < 0.05.

### 2.4 Targeted metabolomics analysis of lung tissue

Metabolites were extracted from homogenized frozen lung tissue samples (25 mg) using the liquid–liquid extraction procedure described previously.^21^, ^22^, ^23^ Pooled mouse liver samples were used for quality control. Deproteinization was performed using a 3K Amico‐Ultra filter (Millipore), followed by drying. Dried pellets were dissolved into methanol–water (50:50 v/v) and subjected to liquid chromatography–tandem mass spectrometry (LC–MS/MS) analysis using hydrophilic interaction chromatography and reverse‐phase chromatography separations.^21^, ^23^ Data were acquired via single reaction monitoring using a 6495 Triple Quadrupole mass spectrometer coupled to an HPLC system (Agilent Technologies) through Agilent Mass Hunter Software. The acquired data from each peak were analyzed and integrated using Agilent Mass Hunter Quantitative Analysis software.

### 2.5 Lung metabolomics analysis

Metabolomics data were normalized to internal standards on a per‐sample, per‐method basis, then log2‐transformed. Differentially expressed metabolites (DEMs) were detected using the unequal variance two‐sided Student's *t*‐test followed by Benjamini‐Hochberg correction, with significance achieved at an FDR‐adjusted *p* < 0.25. Volcano plots were generated using the R statistical system.

### 2.6 Integrative metabolomics and transcriptomics pathway analysis

We determined genes associated with metabolomic signatures using the Human Metabolome Database (HMDB, RRID:SCR_007712).^24^ Enriched pathways were inferred using the ORA method, as implemented by the MSigDB (Molecular Signatures Database, RRID:SCR_016863)^25^ using the hypergeometric test and with significance achieved at an FDR‐adjusted *p* < 0.2.

Differentially co‐expressed modules were detected using the DiffCoEx method.^26^ We analyzed module‐specific hybrid metabolite/transcriptomics pathway using WikiPathways (WikiPathways, RRID:SCR_002134)^27^ with significance achieved at FDR < 0.25.

We used the LUAD integrated transcriptomic/metabolomics signature data to query the Library of Integrated Network‐Based Cellular Signatures (LINCS) for repurposable chemical compounds (LINCS Connectivity Map, RRID:SCR_002639).

### 2.7 Clinical association with TCGA

We determined gene signatures association with patient survival using the TCGA LUAD Cohort. Specimens were stratified and survival association was determined using the R package *survival* ^28^ and the log‐rank test, with significance achieved at *p* < 0.05. Distribution of gene signature score across tumor stages was assessed using the ANOVA test, with significance achieved at *p* < 0.05.

### 2.8 Cell culture and treatments

Normal human bronchial epithelial cells (Beas‐2B) and human LUAD cells (NCI‐H2342) were purchased from ATCC (ATCC Cat# CRL‐9609, RRID: CVCL_0168; ATCC Cat# CRL‐5941, RRID:CVCL_1549). Both cell lines were authenticated from ATCC. Beas‐2B cells were grown in a special media, Bronchial Epithelial Cell Growth Medium Bullet Kit (CC‐3170‐Lonza). H2342 cells were cultured in DMEM:F12 supplemented with 10% fetal bovine serum, 1% penicillin–streptomycin, and 1% glutamine. Cells were seeded at 5000 cells/well in 96‐well plates and grown for 24 h before treatment with various concentrations of AZD‐6482 and PI‐828 (Sigma‐Aldrich, # 1173900‐33‐8 and Tocris‐ 2814/1) for up to 48 h. Cell viability was assessed using the XTT assay.^29^ Absorbance was read at 475 nm with a standard plate reader. The IC_50_ values were calculated using GraphPad Prism software version 8.02 (GraphPad Prism, RRID:SCR_002798). All data in the viability curves are reported as mean ± standard error of the mean.

### 2.9 IPA‐Qiagen

We used Ingenuity pathway analysis (IPA) network analysis (IPA, RRID:SCR_008653) to identify interactions with other genes. We used an ‘enrichment’ score (Fisher's exact test *p*‐value) that measures the overlap of observed and predicted regulated gene sets, and a *Z*‐score assessing the match of observed and predicted up/down‐regulation patterns.

### 2.10 Statistical analysis

We utilized unequal variance two‐sided parametric *t*‐test to assess significance of transcriptomics changes, followed by multiple testing hypothesis corrections using the Benjamini‐Hochberg (False Discovery Rate) method, with significance achieved at FDR < 0.05 and fold change exceeding 1.25X. We utilized unequal variance two‐sided parametric *t*‐test to assess significance of metabolomics changes, followed by Benjamini‐Hochberg correction, with significance achieved for FDR < 0.25. We utilized unequal variance two‐sided parametric *t*‐test with three replicates for Real‐Time PCR analysis, with significance achieved for *p* < 0.05.

## 3 RESULTS

### 3.1 Transcriptomic and metabolic alterations robustly distinguish LUAD and LUSC

The LUAD and LUSC tumors and adjacent normal tissue samples were obtained from both males and females with an age range of 54–80. After removing samples with low‐quality RNA sequencing data, the final analysis included 12 tumors and 13 normal tissues for LUAD and 13 tumors and 12 normal tissues for LUSC as outlined in (Figure 1).

**FIGURE 2:**
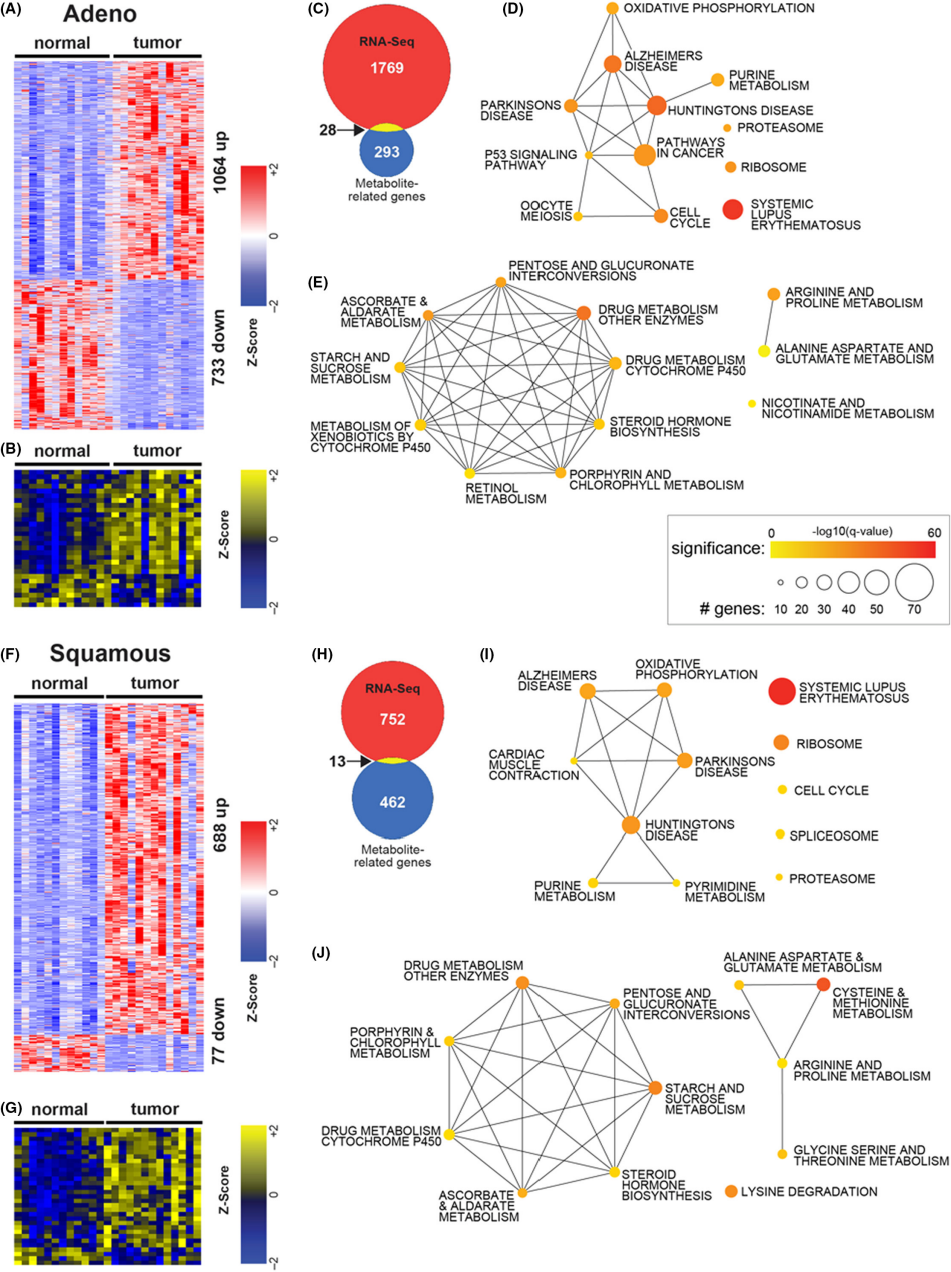
Metabolomic and transcriptomic signatures and enriched pathways in LUAD and LUSC. (A) Heatmap of 1797 genes differentially expressed between tumor and adjacent normal tissue in LUAD (FDR < 0.05, fold change exceeding 1.25X). (B) Heatmap of 29 metabolites differentially expressed between tumor and adjacent normal tissue in LUAD (FDR < 0.25). (C) Venn diagram of 1797 differentially expressed genes and 316 genes associated with differential metabolites in LUAD, yielding an integrative 28‐gene signature. Networks of top enriched pathways using the 1797 differentially expressed genes (D) or 316 genes associated with differentially expressed metabolites (E) in LUAD. (F) Heatmap of 765 genes differentially expressed between tumor and adjacent normal tissue in LUSC (FDR < 0.05, fold change exceeding 1.25X). (G) Heatmap of 33 metabolites differentially expressed between tumor and adjacent normal tissue in LUAD (FDR < 0.25). (H) Venn diagram of 765 differentially expressed genes and 475 gene associated with differential metabolites in LUSC, yielding an integrative 13‐gene signature. Networks of top enriched pathways using the 765 differentially expressed genes (I) or 475 genes associated with differentially expressed metabolites (J) in LUSC. LUAD, lung adenocarcinoma; LUSC, lung squamous cell carcinoma.

We identified 1064 statistically significant upregulated genes and 733 downregulated genes in LUAD versus adjacent normal tissue, and 688 upregulated and 77 downregulated in LUSC versus adjacent normal tissue (Figure 2A,F). Overlap of the two signatures showed 329 upregulated and 30 downregulated genes in common between LUAD and LUSC (Figures S1–S8). Targeted metabolomics identified DEMs in each tumor subtype (Figure 2B,G and Figure S2). We mapped the altered metabolites to their corresponding genes using the HMDB, resulting in 293 LUAD and 462 LUSC altered metabolite‐related genes. Integration of these two omics platforms identified 28 intersecting genes for LUAD and 13 for LUSC (Figure 2C,H). Only three metabolite‐related genes were common between LUAD and LUSC (Figure S3). To further explore altered biochemical pathways, we performed pathway enrichment analysis of the metabolite‐related genes and identified 39 significantly enriched biochemical pathways (*p* < 0.05). Of these pathways, 24 were highly interconnected and related to the synthesis of amino acids, namely lysine, cysteine, methionine, arginine, proline, tryptophan, and alanine as well as glycolysis, lipid metabolism, and fatty acids (Figure 2D,E,I,J). These results highlight differences between LUAD and LUSC in terms of differential expression of genes, altered metabolites, and dysregulation of biochemical pathways.

### 3.2 Metabolic networks are disrupted during LUAD and LUSC tumorigenesis

**FIGURE 3:**
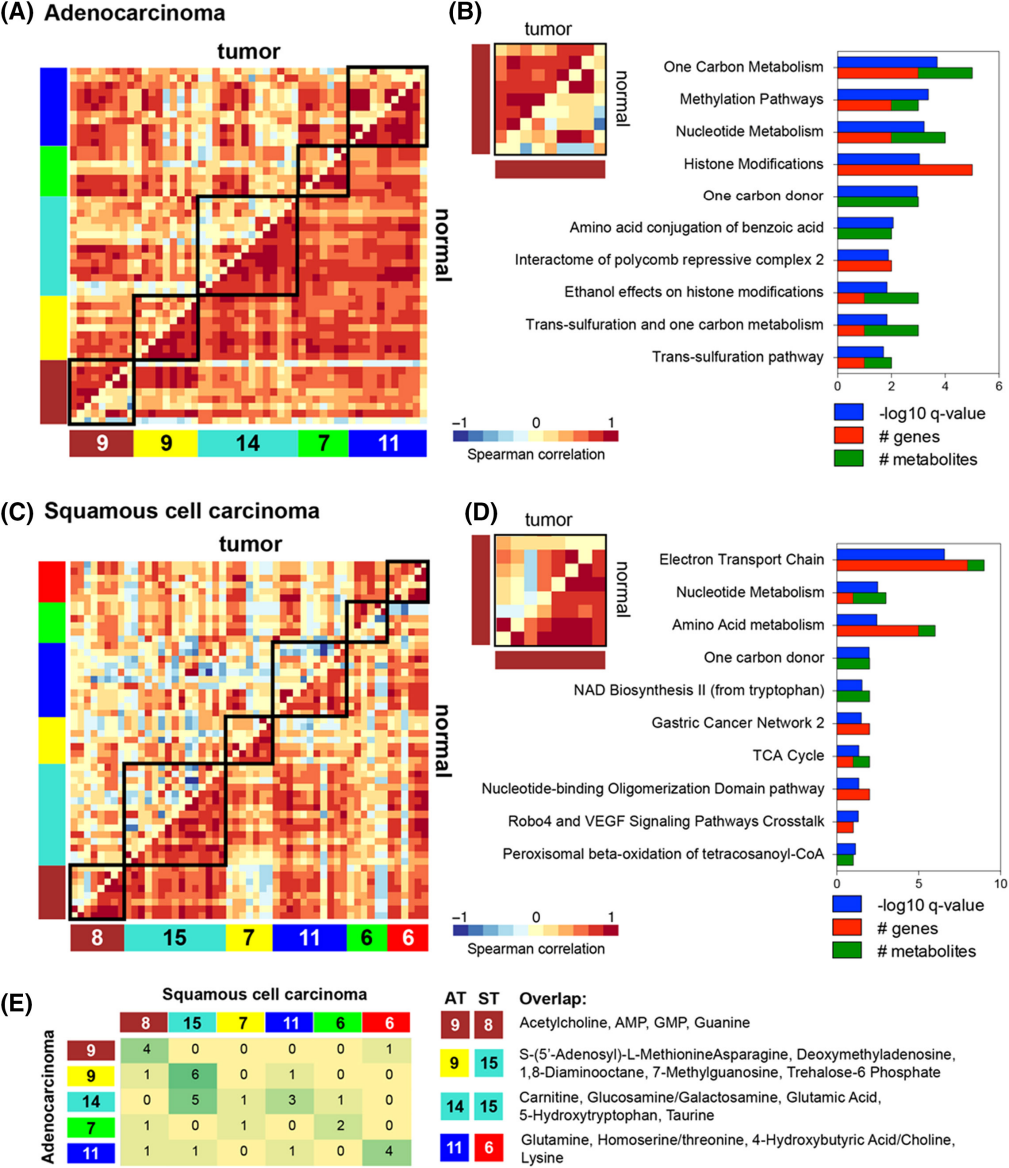
Differential co‐expression analysis in LUAD and LUSC metabolites. (A) DiffCoEx identified five modules of metabolites differentially correlated between LUAD tumor and adjacent normal samples. The brown module, with nine metabolites, contains metabolites with increased correlation in tumor samples. (B) Top 10 enriched pathways in LUAD using metabolites from the brown module and associated differentially expressed genes, graphed as −log10 *p*‐value, showing also the number of metabolites or metabolite‐related genes contributing to pathway enrichment. Significance was determined by hypergeometric distribution. (C) Differential co‐expression analysis identified six modules of metabolites differentially correlated between LUSC tumor and adjacent normal samples. The green module, including six metabolites, contains metabolites with increased correlation in tumor samples. (D) Top 10 enriched pathways in LUSC using metabolites from the brown module and associated differentially expressed genes, graphed as −log10 *p*‐value, showing also the number of metabolites or metabolite‐related genes contributing to pathway enrichment. Significance was determined by hypergeometic distribution. (E) Pairwise overlap analysis between the LUAD and LUSC modules shows both similar and distinct patterns of metabolite differential co‐expression between the two cancer types. DiffCoEx, differential co‐expression analysis; LUAD, lung adenocarcinoma; LUSC, lung squamous cell carcinoma.

To identify disruption of existing biological processes or gain of new oncogenic processes during tumorigenesis, we used Differential CoExpression (DiffCoEx) analysis. The number and direction of DEMs associated with each DiffCoEx module are presented in Figure S4; the majority of metabolites in DiffCoEx modules do not overlap with DEMs. Using the metabolites expressed in tumor compared to adjacent normal, we observed loss of correlation between metabolites in both LUAD (Figure 3A) and LUSC (Figure 3C) tumors, reflecting disruption of normal lung function. However, the brown module in LUAD showed a gain of correlation in one group of nine metabolites (Figure 3B), suggestive of development of an onco‐metabolomic module. We combined these nine metabolites with the 57 metabolite‐related genes from the RNA‐seq analysis and performed over‐representation analysis of pathway enrichment (Figure 3B), revealing a significant portion were associated with one‐carbon metabolism and methylation pathways. The corresponding brown module in LUSC showed loss of coherence in cancer and by combining its eight metabolites with the 42 metabolite‐related genes (Figure 3D) found that genes were associated with electron transport chain and nucleotide metabolism pathways. Pathway enrichment results for the other modules are shown in Figures S5 (LUAD) and S6 (LUSC). In a pairwise overlap analysis of all modules identified in each subtype (Figure 3E), we identified shared DEMs between LUAD and LUSC. Four of the eight metabolites from the LUSC brown module were shared with the onco‐metabolic LUAD brown module (4 out of 9), including GMP, guanine, AMP, and acetylcholine. The LUSC turquoise module (15 metabolites) had six and five metabolites in common with the LUAD yellow and turquoise models, respectively. Finally, 4 out of 6 metabolites in the LUSC red module overlapped with 4 out of 11 metabolites in the LUAD blue module. These results show that there are unique metabolite modules and related tumor gene signatures that discriminate LUAD from LUSC, but also previously unappreciated common metabolic networks, indicating potential biomarkers and novel therapeutic vulnerabilities between the two subtypes.

### 3.3 Integrative metabolomic/transcriptomic signature has prognostic capabilities in LUAD

**FIGURE 4:**
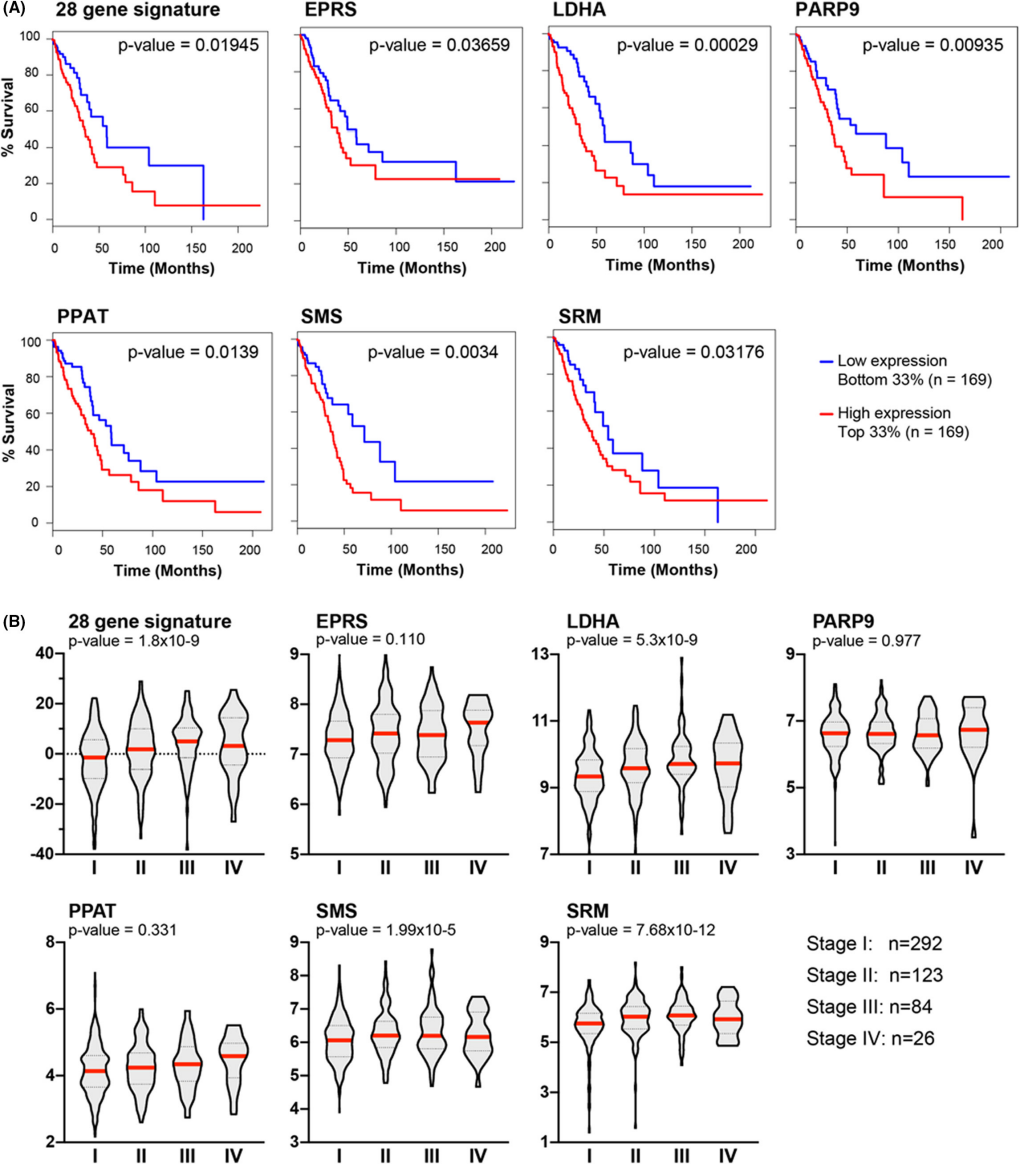
Clinical associations of the LUAD integrative transcriptomics and metabolomics signature in the TCGA LUAD cohort. (A) Survival analysis indicates that patients in the top tertile for activity score of the integrative 28‐gene signature have lower prognosis compared to those in the lower tertile (log‐rank test, *p* < 0.05). Six individual genes in the 28‐gene signature, EPRS, LDHA, PARP9, PPAT, SMS, and SRM, show similar prognosis as the full signature. (B) The 28‐gene signature shows increased activity score with increased tumor stage in the TCGA LUAD cohort (ANOVA test, *p* < 0.05). Similar properties are observed for 3 out of the 28 individual genes, LDHA, SMS, and SRM. LUAD, lung adenocarcinoma; TCGA, The Cancer Genome Atlas.

Our multi‐modal integration of metabolomic and transcriptomic signatures revealed 28 common genes in LUAD (Figure 2C). Intriguingly, this signature was significantly associated with survival in the TCGA LUAD cohort, with a higher activity score associated with a worse prognosis (log‐rank test, *p* < 0.02). Because the LUSC 13‐gene signature was not associated with prognosis in the TCGA LUSC cohort, we elected to proceed with further analysis only in the LUAD cohort. Survival analysis in LUAD, based on individual genes, identified six upregulated genes (EPRS, LDHA, PARP9, PPAT, SMS, and SRM) significantly associated with survival (Figure 4A). In addition, we analyzed whether the 28‐gene signature or component genes were associated with different stages of lung cancer. We observed that the activity score of the 28‐gene signature, and expression of three of the six genes associated with survival (LDHA, SMS, and SRM), showed a significant, increasing association with advancing LUAD stages (I–IV) (ANOVA, *p* < 0.05) (Figure 4B). Together, these results show that the integrated transcriptomic/metabolomic gene signature, as well as the six upregulated genes, may be potentially be used as biomarkers to predict patient survival.

### 3.4 The 28‐gene multi‐omics signature can be used to identify chemoprevention modalities

**FIGURE 5:**
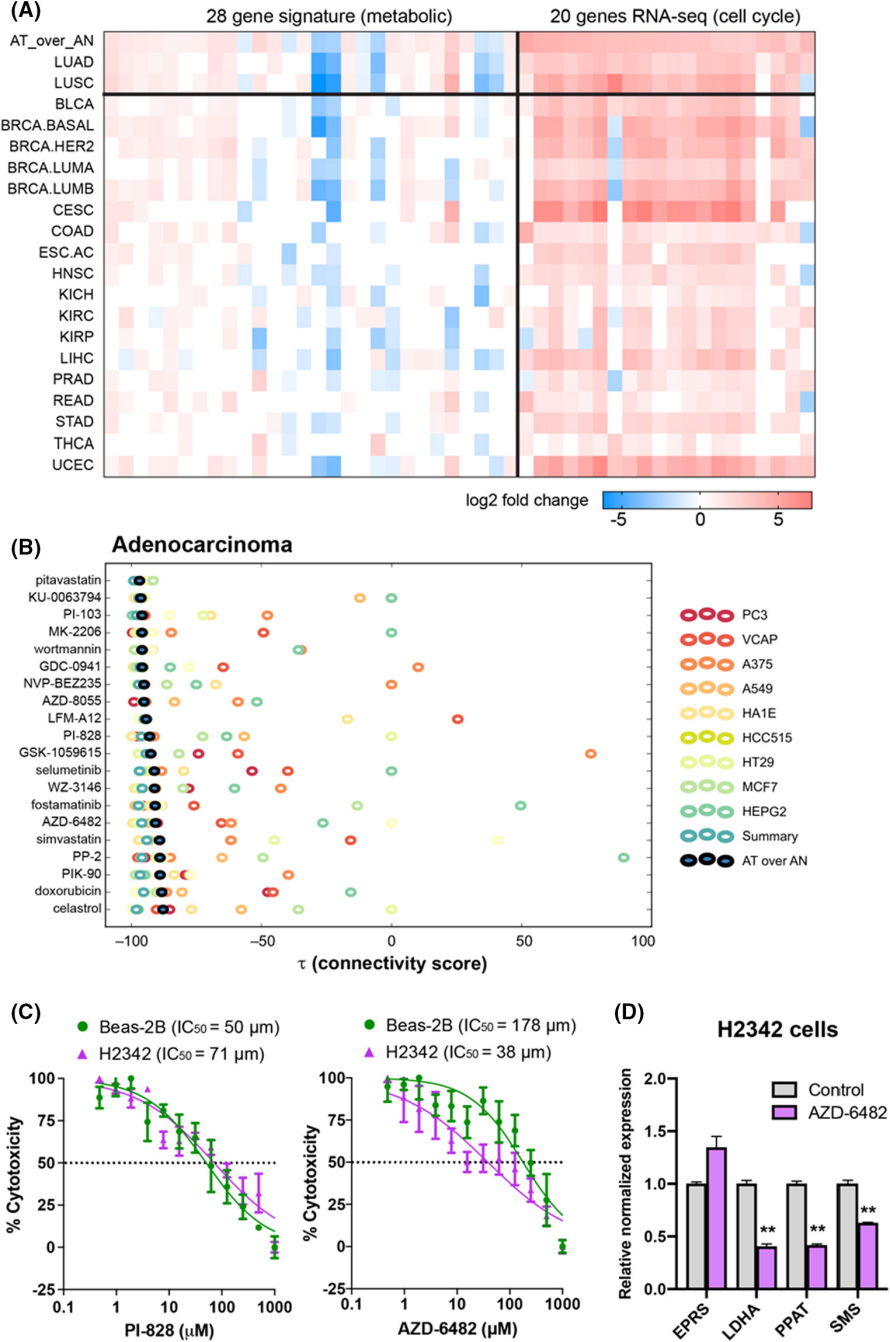
Integrative transcriptomic/metabolomics signature highlights therapeutic vulnerabilities in LUAD. (A) Heatmap showing fold changes for the integrative 28‐gene signature and for 20 differentially expressed genes involved in the cell cycle pathway across 20 TCGA cancer cohorts. Whereas the cell cycle genes are differentially expressed and increased across the majority of the cancers, the integrative transcriptomic/metabolomic gene signature shows cancer‐specific patterns. (B) Top 20 repurposable chemical compounds based on LINCS/Connectivity Map, targeting the integrative 28‐gene signature. The final connectivity score of each compound (indicated by black circles) is based on the integration of connectivity scores of the same chemical compound over a diverse collection of cancer cell lines. (C) IC_50_ analysis of PI‐828 or AZD‐6482, potential LUAD drugs identified in (B) and targeting the dysregulated transcriptomic/metabolic axis. PI‐828 shows no significant difference in IC50 between the two cell lines, while AZ‐6482 shows a higher IC_50_ in Beas‐2B normal cells (178 μM) compared to H2342 LUAD cells (38 μM). (D) RT‐PCR analysis of H2342 cells treated with AZD‐6482 shows that expression of putative oncogenes LDHA, PPAT, and SMS is reduced (*n* = 3 replicates, **p < 0.01). LUAD, lung adenocarcinoma; TCGA, The Cancer Genome Atlas.

Because the genes in the 28‐gene LUAD signature showed distinct patterns across the major cancer types compared to consistently upregulated genes, such as those associated with the cell cycle (Figure 5A), we hypothesized the metabolite‐related signature could be used to identify chemopreventive agents. We queried the LINCS/Connectivity Map resource, which evaluates chemical compound profiles across multiple cancer cell lines and computes an aggregated similarity score. Out of the top 20 chemopreventive agents ranked by anti‐correlation score with the 28‐gene signature (Figure 5B), 16 (80%) were kinase inhibitors. Fourteen of the compounds have been used in clinical trials (according to clinicaltrials.gov), and seven of those have been used for NSCLC. Of the eight PI3K inhibitors, only one had been used in a NSCLC clinical trial (GDC‐0941/pictilisib) and four others had been used in clinical trials for other diseases. We selected two drugs, AZD‐6482 (PI3Kβ inhibitor) and PI‐828 (PI3K inhibitor), for further study. AZD‐6482 has been used in two clinical trials to test an anti‐platelet effect, while PI‐828 has not been used in humans. In a cytotoxicity assay, PI‐828 inhibited both H2342 lung cancer cells and Beas‐2B normal lung epithelial cells similarly, with IC_50_ values of 71 and 50 μM respectively (Figure 5C). In contrast, AZD‐6482 inhibited the growth of H2342 cells, with an IC_50_ value of 40 μM compared to 178 μM for Beas‐2B cells. Since AZD‐6482 inhibited H2342 cells at a lower concentration with negligible effect on normal lung epithelial cells (Beas‐2B), we proceeded with this drug for further experiments. To determine potential therapeutic targets, we selected six genes with differences in LUAD survival, along with a few other genes from the 28 gene signature and six cell cycle‐specific genes from the RNA‐Seq data (see Figure S7). Cell cycle genes were included because deregulation of cell cycle events is common in NSCLC, and various inhibitors of AURKB kinases are being evaluated for potential targeted therapy in NSCLC.^30^ Using RT‐PCR, we observed that AZD‐6482 significantly inhibited expression of three tumor survival markers, LDHA, PPAT, and SMS (*t*‐test, *p* < 0.05) (Figure 5D). Notably, LDHA was found to interact with the cell cycle‐related PLK1‐AURKB‐CENPA axis (see Figure S8). Our data suggest that AZD‐6482 could potentially suppress three metabolic tumor survival markers to potentially increase the survival of LUAD cancer patients.

## 4 DISCUSSION

The rapid development of omics technology has facilitated molecular insights into NSCLC. These studies have revealed transcriptomic expression signatures associated with clinical outcomes in NSCLC,^17^, ^31^, ^32^, ^33^, ^34^, ^35^ protein signatures with biomarker potential,^16^, ^36^ and epigenomics markers revealing changes in LUAD and LUSC that could be useful prognostic biomarkers for cancer progression or response to treatment.^7^, ^8^, ^37^, ^38^ Multi‐omics integrative analysis, which aims to expand the understanding of carcinogenesis from a single parameter model to a systemic multi‐parameter model, has enabled subtype identification and characterization in both LUAD and LUSC.^5^, ^6^, ^39^ However, the limited number of LUAD and LUSC studies integrating transcriptomics or proteomics with metabolomics have not generated lung cancer biomarkers or identified novel anti‐cancer drugs targeting the disrupted metabolic/transcriptomic networks. To fill this knowledge gap, we integrated transcriptomics with metabolomics in a systems biology approach to reveal metabolite‐related gene signatures, altered metabolic pathways, potential prognostic markers, and metabolite gene therapeutic targets in LUAD and LUSC.

Previous studies identified individual metabolism‐associated genes associated with survival, such as ATIC and ADSL, which are associated with purine metabolism.^40^ Based on our experience identifying metabolism‐associated transcriptomic or proteomic markers in other cancers,^22^, ^41^, ^42^, ^43^ we sought to identify metabolite‐related genes as potential prognostic markers for LUAD and LUSC. We have identified a 28‐gene metabolite‐related signature for LUAD and a 13‐gene signature for LUSC. High expression of these 28 LUAD‐specific genes, but not of the 13 LUSC‐specific genes, was associated with poor survival in the TCGA LUAD dataset. We analyzed the prognostic capability of individual genes from the LUAD‐specific 28‐gene signature and found that higher expression of six genes, including EPRS, LDHA, PARP9, PPAT, SMS, and SRM, was associated with poor survival. Recent studies demonstrated that aberrant expression of these genes at the transcript or protein level was associated with lung malignancies and the genes were potential therapeutic targets.^16^, ^36^, ^44^, ^45^, ^46^ Tumor stage is currently utilized as a strong indicator of survival in NSCLC,^47^ but is not very accurate at the individual level.^48^ Our analysis showed that expression of the LUAD‐specific 28‐gene signature, as well as that of three of the six survival‐associated genes (LDHA, SMS, and SRM), significantly increased from Stage I to Stage IV in the TCGA data set. Our study provides both individual genes and complex gene signatures that could serve as prognostic biomarkers in LUAD to improve therapeutic decisions.

Recent studies and ongoing trials have aimed to identify novel metabolism‐targeting drugs. To determine whether the LUAD‐specific 28‐gene signature could contain novel therapeutic targets, we used LINCS/Connectivity Map analysis^49^, ^50^ to rank repurposable drugs with potential novel efficacy against lung cancer. AZD‐6482, a drug that completed Phase 1 clinical trials for antiplatelet effects (NCT00688714, NCT00853450), was selected for further evaluation. AZD‐6482 is a selective PI3K p110β inhibitor and the PI3K signaling pathway is highly critical in a variety of human cancers, including NSCLC.^51^ Therefore, targeted PI3K inhibitors are potential anticancer drugs. Previous studies have reported that p110β may play a pivotal role in PTEN loss‐induced tumorigenesis in glioblastoma.^52^ Loss of PTEN expression is a frequent occurrence in lung cancer, regulated at many levels.^53^ Here, we examined the effect of AZD‐6482 on expression of the 28 genes from the metabolite‐related signature, as well as six cell cycle genes and 12 other differentially expressed genes, to determine potential therapeutic targets. Three metabolite‐related genes (LDHA, PPAT, and SMS) were increased in LUAD tumors but markedly suppressed after treatment with AZD‐6482. LDHA has also previously been shown to be upregulated in LUAD, but not LUSC, and has potential as a prognostic indicator of poor survival.^46^ Herein, we showed for the first time that a p110β‐selective inhibitor, AZD6482, could inhibit metabolite‐related genes which are upregulated in many cancer cells, including LUAD and LUSC.

IPA analysis identified that LDHA interacts with the PLK1‐AURKB‐CENPA axis. PPAT is also strongly correlated with malignancy, particularly in neuroendocrine cancer including small cell lung cancer (SCLC), and its depletion suppresses the growth of SCLC lines.^45^ SMS, a polyamine biosynthetic enzyme with no reported role in NSCLC thus far, is overexpressed in colorectal cancer and its disruption leads to accumulation of spermidine, which induces the expression of proapoptotic protein Bim.^54^ These potential therapeutic targets for a repurposed drug provide novel insight into the potential treatment of LUAD by inhibiting enzymatic activity or otherwise exploiting these metabolic pathways.

Integration of transcriptomic and metabolomic data can reveal metabolic reprogramming signatures and significantly enriched metabolic pathways associated with LUSC and LUAD, as was recently reported in a weighted gene co‐expression network analysis.^55^ We conducted DiffCoEx analysis and observed that both LUAD and LUSC are characterized by loss of correlation of metabolites in tumors compared to normal adjacent tissues, indicative of the disruption of normal tissue biology. However, we also identified an onco‐metabolic module with increased inter‐metabolite correlation in LUAD tumors, associated with metabolic pathways including methylation, one‐carbon metabolism, nucleotide metabolism, and histone modifications. Notably, LUSC exhibited a significant loss of correlation of four metabolites from the corresponding module, which was enriched in electron transport chain and amino acid metabolism pathways. Our results demonstrate that differential co‐expression is a powerful approach to distinguish NSCLC subtypes. The metabolic differences between subtypes appear to result from systemic molecular changes driven by both altered metabolic and aberrant signaling pathways.

In conclusion, for the first time, multi‐omics integration of transcriptomics and metabolomics was used to identify unique metabolite gene signatures specifically for LUAD and LUSC, and prognostic markers and therapeutic targets for LUAD. We further provided a proof of concept that repurposed drugs targeting transcriptional programs associated with metabolic dysregulation may have anti‐tumor effects in LUAD. Ultimately, the metabolite‐related gene signatures and altered metabolic pathways may offer novel insights into the prognosis and innovative treatment of NSCLC.

## AUTHOR CONTRIBUTIONS

All authors of this research paper have directly participated in the planning, execution, and/or analysis of this study. Conceptualization, Jose Thaiparambil, Cristian Coarfa, and Randa El‐Zein; Data curation, Jose Thaiparambil, Jianrong Dong, Sandra L. Grimm, Dimuthu Perera, Matthew J. Robertson, Brandon Mistretta, Preethi H. Gunaratne, Nagireddy Putluri, Cristian Coarfa, Randa El‐Zein; Formal analysis, Jianrong Dong, Sandra L. Grimm, Dimuthu Perera, Tajhal D. Patel, Nagireddy Putluri; Funding acquisition, Preethi H. Gunaratne, Nagireddy Putluri, Cristian Coarfa, Randa El‐Zein; Investigation, all authors; Methodology, all authors; Project administration, Jose Thaiparambil, Cristian Coarfa, Randa El‐Zein; Resources, Jose Thaiparambil, Jianrong Dong, Sandra L. Grimm, Dimuthu Perera, Matthew J. Robertson, Preethi H. Gunaratne, Min P. Kim, Nagireddy Putluri, Cristian Coarfa, Randa El‐Zein; Software, Jianrong Dong, Dimuthu Perera, Matthew J. Robertson, Cristian Coarfa; Supervision, Jose Thaiparambil, Preethi H. Gunaratne, Jason T. Yustein, Nagireddy Putluri, Cristian Coarfa, Randa El‐Zein; Validation, Jose Thaiparambil, Jianrong Dong, Sandra L. Grimm, Dimuthu Perera, Nagireddy Putluri, Cristian Coarfa, Randa El‐Zein; Visualization, Jose Thaiparambil, Jianrong Dong, Sandra L. Grimm, Dimuthu Perera, Cristian Coarfa, Randa El‐Zein; Writing—original draft preparation, Jose Thaiparambil, Sandra L. Grimm, Cristian Coarfa, Randa El‐Zein; Writing—review and editing, Jose Thaiparambil, Sandra L. Grimm, Preethi H. Gunaratne, Jason T. Yustein, Nagireddy Putluri, Cristian Coarfa, Randa El‐Zein.

## FUNDING INFORMATION

This project was partially supported by The Cancer Prevention Institute of Texas (CPRIT) grants RP170005, RP210227, and RP200504, NIH P30 shared resource grant CA125123, and NIEHS grants P30 ES030285 and P42 ES027725.

## CONFLICT OF INTEREST

The authors declare no conflicts of interest.

## ETHICS APPROVAL AND CONSENT TO PARTICIPATE

The study protocol was approved by the institutional Review Board of the Houston Methodist Research Institute (Pro00014471).

## DISCLAIMER

All analyses and conclusions in this manuscript are the sole responsibility of the authors and do not necessarily reflect the opinions or views of CPRIT, NIH, NIEHS, or the authors' institutions.

## CONSENT FOR PUBLICATION

Written informed consent was obtained from all patients before enrollment.

## Supporting information


Figure S1–S8

## Data Availability

The RNA‐Seq data underlying this article are available in the NCBI Gene Expression Omnibus at http://www.ncbi.nlm.nih.gov/geo/ and can be accessed with accession number GSE159857. The metabolomics data underlying this article are available on reasonable request to the corresponding author.
