# Supplementary material for: Integrative metabolomics and transcriptomics analysis reveals novel therapeutic vulnerabilities in lung cancer
Source: Cancer Med. 2022 Jun 8;12(1):584–96. doi: 10.1002/cam4.4933 (PMC9844651; doi:10.1002/cam4.4933)

Figure 1

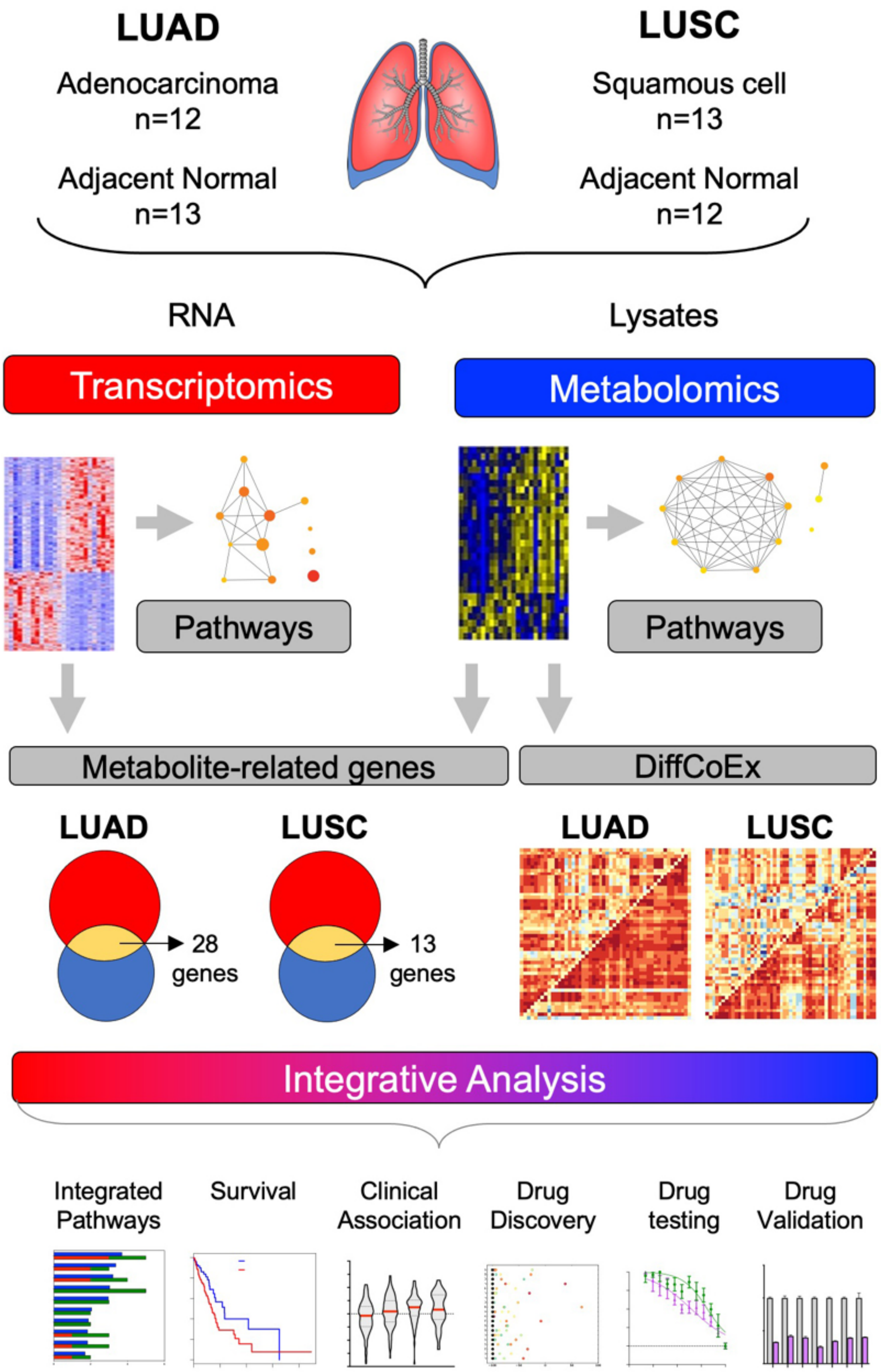
LUAD
Adenocarcinoma
n=12
Adjacent Normal
n=13
LUSC
Squamous cell
n=13
Adjacent Normal
n=12
RNA
Lysates
Transcriptomics
Metabolomics
Pathways
Pathways
Metabolite-related genes
DiffCoEx
LUAD
LUSC
28
genes
13
genes
LUAD
LUSC
Integrative Analysis
Integrated Pathways
Survival
Clinical Association
Drug Discovery
Drug testing
Drug Validation

Figure 2

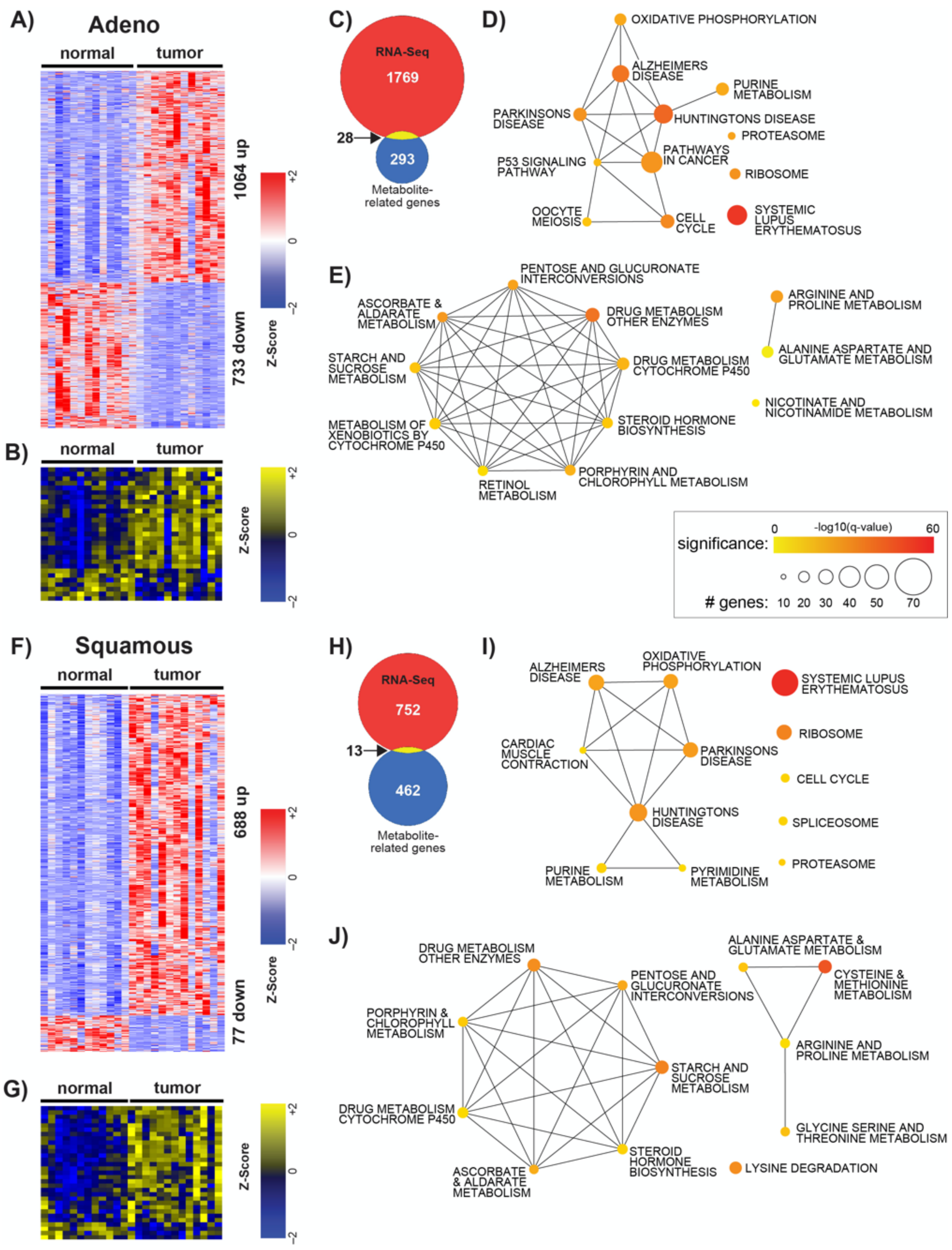

A)
Adeno
normal
tumor
1064 up
733 down
Z-Score
+2
0
-2
B)
normal
tumor
Z-Score
C)
RNA-Seq
1769
28
293
Metabolite-related genes
D)
OXIDATIVE PHOSPHORYLATION
ALZHEIMERS DISEASE
PURINE METABOLISM
PARKINSONS DISEASE
HUNTINGTONS DISEASE
PROTEASOME
PATHWAYS IN CANCER
P53 SIGNALING PATHWAY
RIBOSOME
OOCYTE MEIOSIS
CELL CYCLE
SYSTEMIC LUPUS ERYTHEMATOSUS
E)
PENTOSE AND GLUCURONATE INTERCONVERSIONS
ASCORBATE & ALDARATE METABOLISM
DRUG METABOLISM OTHER ENZYMES
ARGININE AND PROLINE METABOLISM
ALANINE ASPARTATE AND GLUTAMATE METABOLISM
STARCH AND SUCROSE METABOLISM
DRUG METABOLISM CYTOCHROME P450
NICOTINATE AND NICOTINAMIDE METABOLISM
METABOLISM OF XENOBIOTICS BY CYTOCHROME P450
STEROID HORMONE BIOSYNTHESIS
RETINOL METABOLISM
PORPHYRIN AND CHLOROPHYLL METABOLISM
significance:
0
-log10(q-value)
60
genes:
10 20 30 40 50 70
F)
Squamous
normal
tumor
688 up
77 down
Z-Score
G)
normal
tumor
Z-Score
H)
RNA-Seq
752
13
462
Metabolite-related genes
I)
ALZHEIMERS DISEASE
OXIDATIVE PHOSPHORYLATION
SYSTEMIC LUPUS ERYTHEMATOSUS
RIBOSOME
CARDIAC MUSCLE CONTRACTION
PARKINSONS DISEASE
CELL CYCLE
HUNTINGTONS DISEASE
SPLICEOSOME
PURINE METABOLISM
PYRIMIDINE METABOLISM
PROTEASOME
J)
DRUG METABOLISM OTHER ENZYMES
ALANINE ASPARTATE & GLUTAMATE METABOLISM
PENTOSE AND GLUCURONATE INTERCONVERSIONS
CYSTEINE & METHIONINE METABOLISM
PORPHYRIN & CHLOROPHYLL METABOLISM
ARGININE AND PROLINE METABOLISM
STARCH AND SUCROSE METABOLISM
DRUG METABOLISM CYTOCHROME P450
GLYCINE SERINE AND THREONINE METABOLISM
STEROID HORMONE BIOSYNTHESIS
ASCORBATE & ALDARATE METABOLISM
LYSINE DEGRADATION

Figure 3

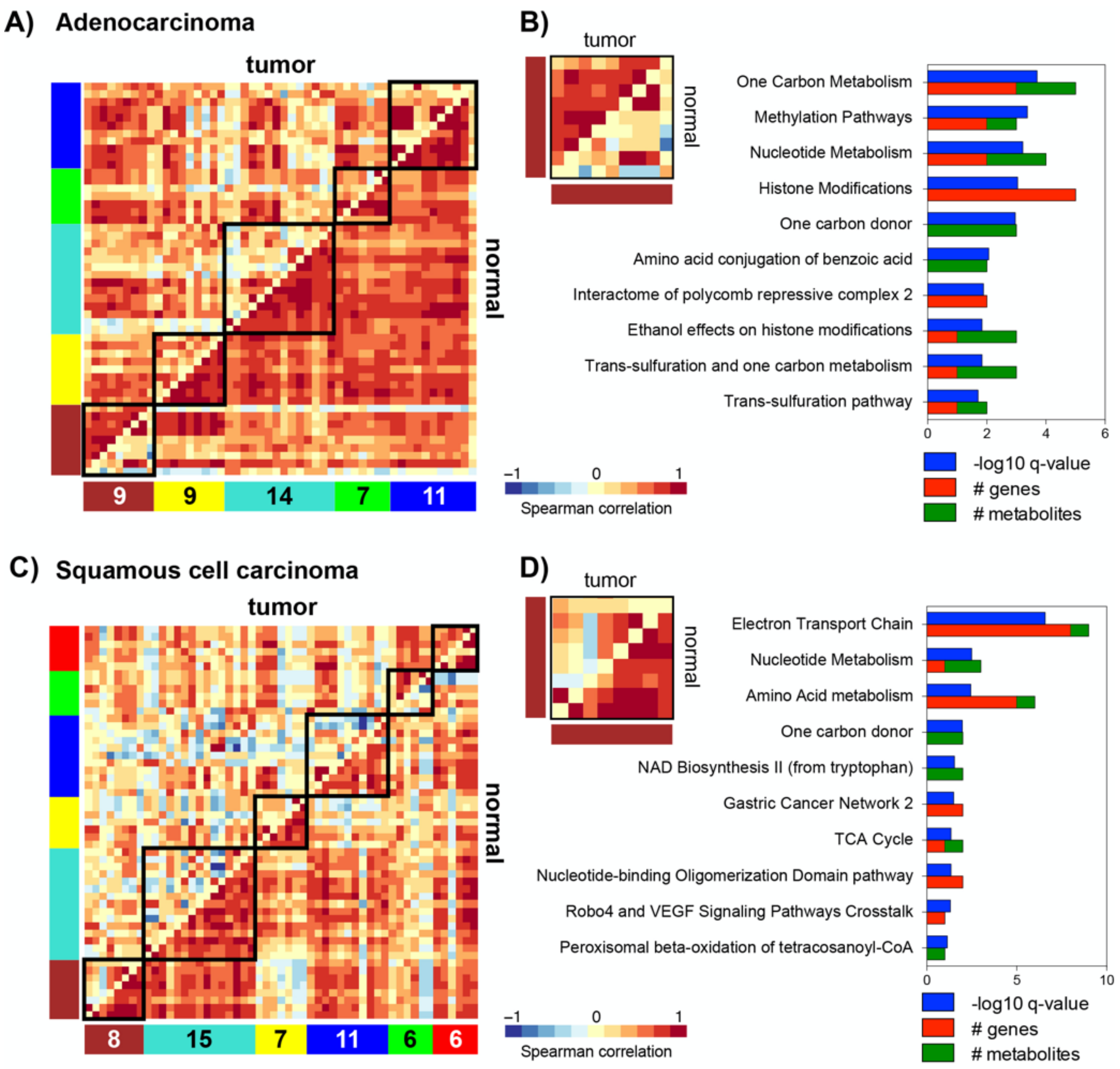

E)

Squamous cell carcinoma (columns) vs. Adenocarcinoma (rows)

Adenocarcinoma	8	15	7	11	6	6
9	4	0	0	0	0	1
9	1	6	0	1	0	0
14	0	5	1	3	1	0
7	1	0	1	0	2	0
11	1	1	0	1	0	4

AT	ST	Overlap:
9	8	Acetylcholine, AMP, GMP, Guanine
9	15	S-(5'-Adenosyl)-L-MethionineAsparagine, Deoxymethyladenosine, 1,8-Diaminooctane, 7-Methylguanosine, Trehalose-6 Phosphate
14	15	Carnitine, Glucosamine/Galactosamine, Glutamic Acid, 5-Hydroxytryptophan, Taurine
11	6	Glutamine, Homoserine/threonine, 4-Hydroxybutyric Acid/Choline, Lysine

Figure 4

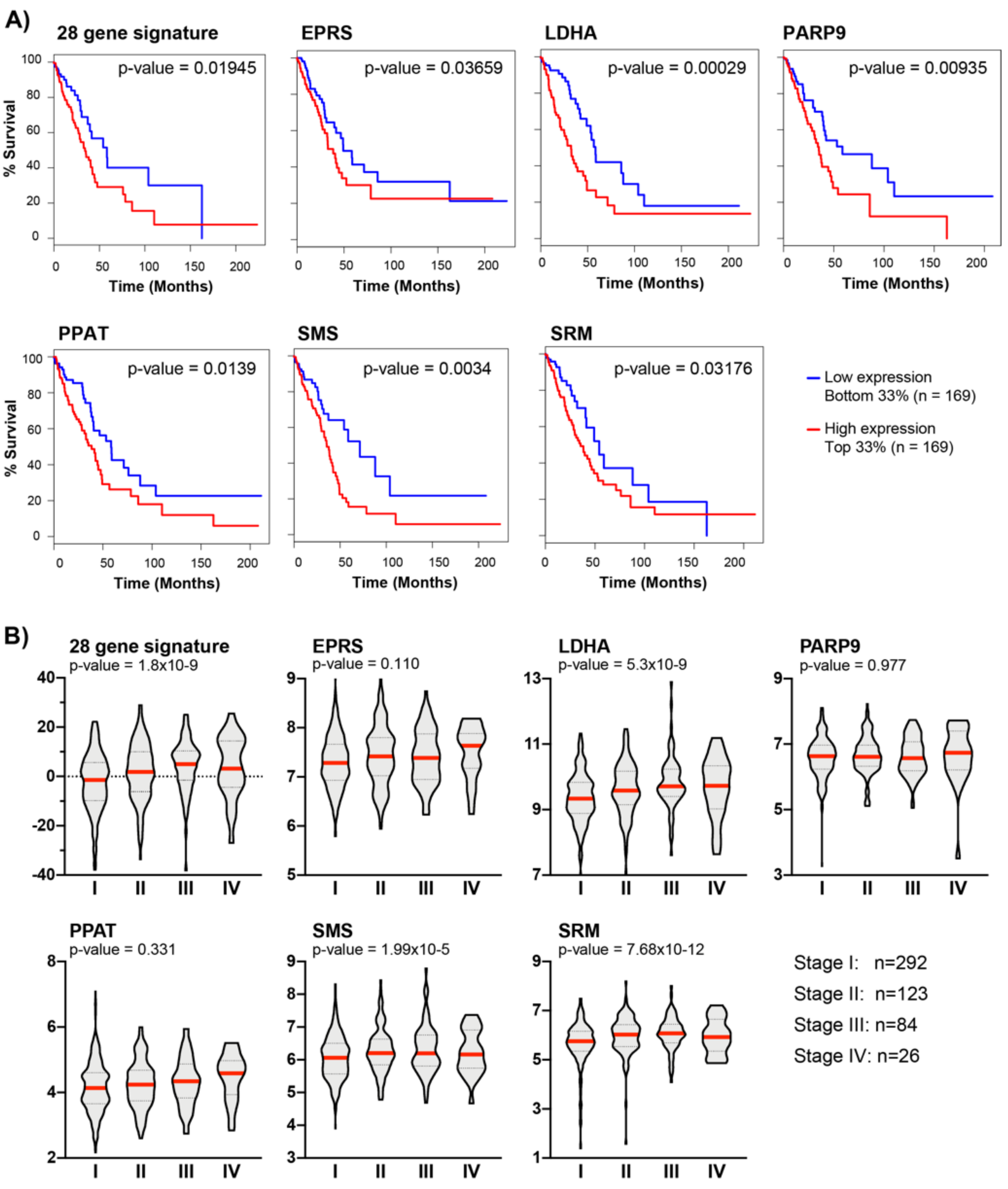
A)
28 gene signature
p-value = 0.01945
EPRS
p-value = 0.03659
LDHA
p-value = 0.00029
PARP9
p-value = 0.00935
PPAT
p-value = 0.0139
SMS
p-value = 0.0034
SRM
p-value = 0.03176
% Survival
Time (Months)
Low expression
Bottom 33% (n = 169)
High expression
Top 33% (n = 169)
B)
28 gene signature
p-value = 1.8x10-9
EPRS
p-value = 0.110
LDHA
p-value = 5.3x10-9
PARP9
p-value = 0.977
PPAT
p-value = 0.331
SMS
p-value = 1.99x10-5
SRM
p-value = 7.68x10-12
I
II
III
IV
Stage I: n=292
Stage II: n=123
Stage III: n=84
Stage IV: n=26

Figure 5

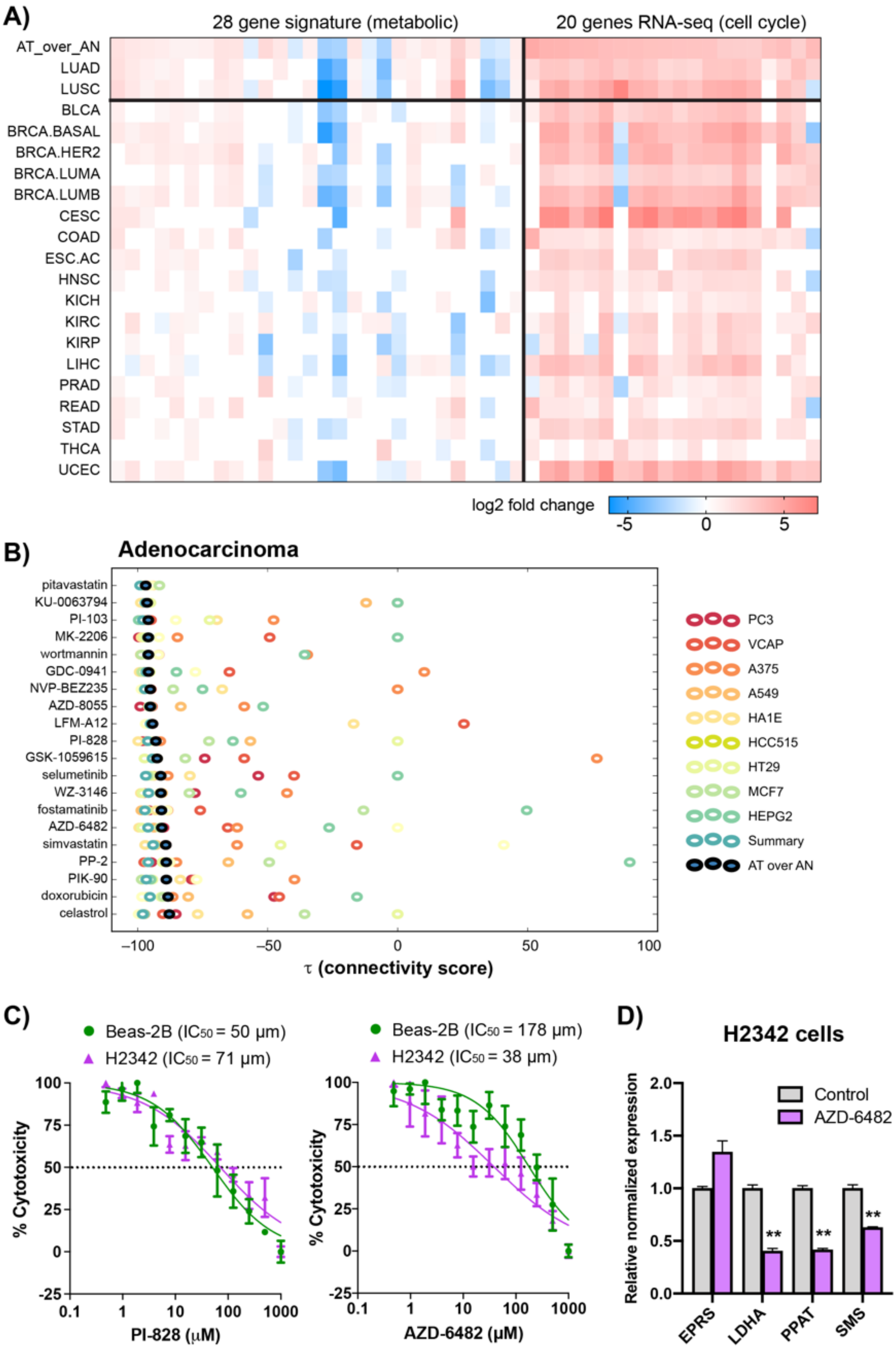

A)
28 gene signature (metabolic)
20 genes RNA-seq (cell cycle)
AT_over_AN
LUAD
LUSC
BLCA
BRCA.BASAL
BRCA.HER2
BRCA.LUMA
BRCA.LUMB
CESC
COAD
ESC.AC
HNSC
KICH
KIRC
KIRP
LIHC
PRAD
READ
STAD
THCA
UCEC
log2 fold change
-5
0
5
B)
Adenocarcinoma
pitavastatin
KU-0063794
PI-103
MK-2206
wortmannin
GDC-0941
NVP-BEZ235
AZD-8055
LFM-A12
PI-828
GSK-1059615
selumetinib
WZ-3146
fostamatinib
AZD-6482
simvastatin
PP-2
PIK-90
doxorubicin
celastrol
–100
–50
0
50
100
τ (connectivity score)
PC3
VCAP
A375
A549
HA1E
HCC515
HT29
MCF7
HEPG2
Summary
AT over AN
C)
Beas-2B (IC50 = 50 μm)
H2342 (IC50 = 71 μm)
% Cytotoxicity
100
75
50
25
0
-25
0.1
1
10
100
1000
PI-828 (μM)
Beas-2B (IC50 = 178 μm)
H2342 (IC50 = 38 μm)
% Cytotoxicity
100
75
50
25
0
-25
0.1
1
10
100
1000
AZD-6482 (μM)
D)
H2342 cells
Relative normalized expression
2.0
1.5
1.0
0.5
0.0
Control
AZD-6482
**
**
**
EPRS
LDHA
PPAT
SMS

Supp Figure 1

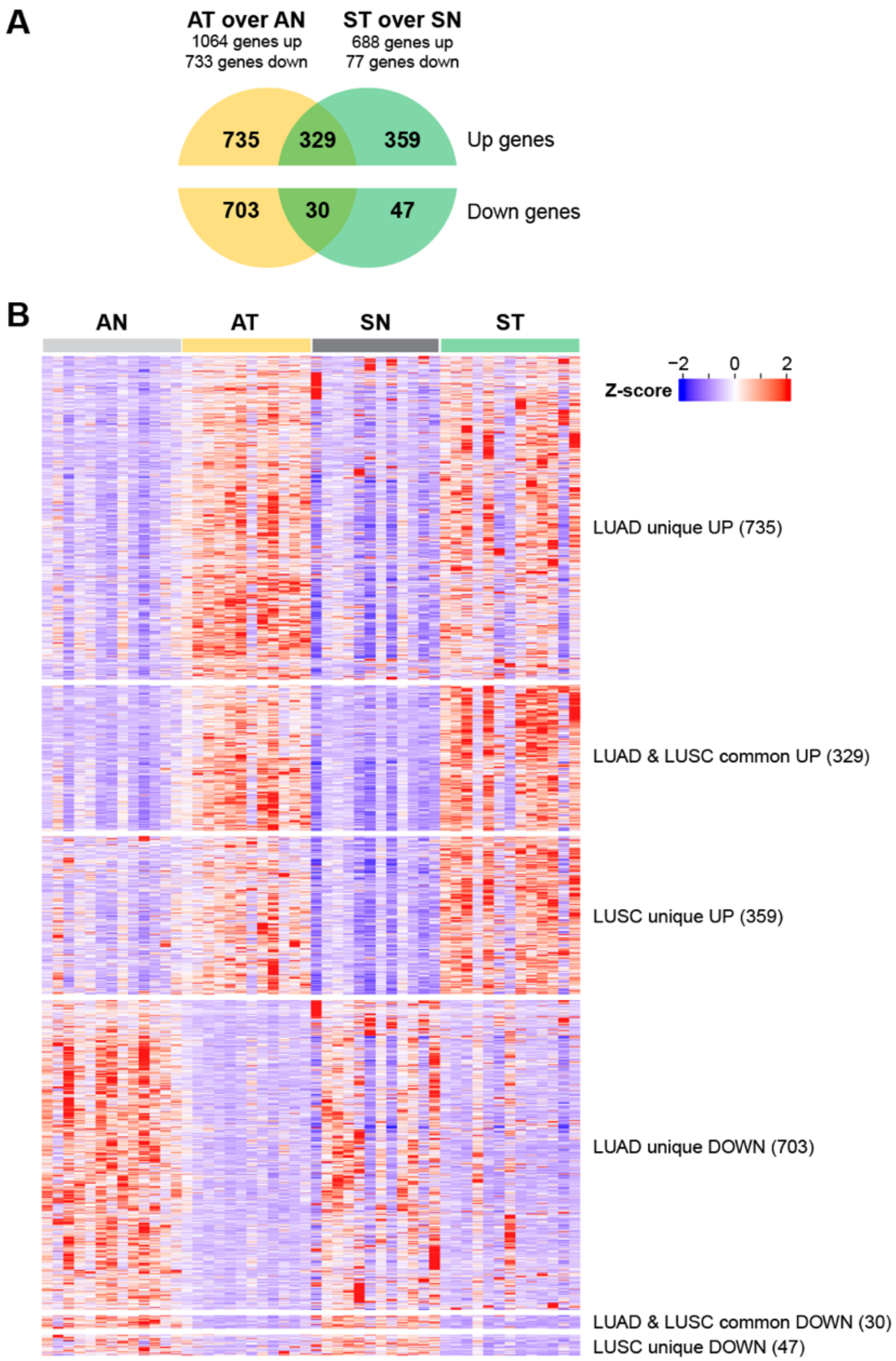

Supp Figure 2

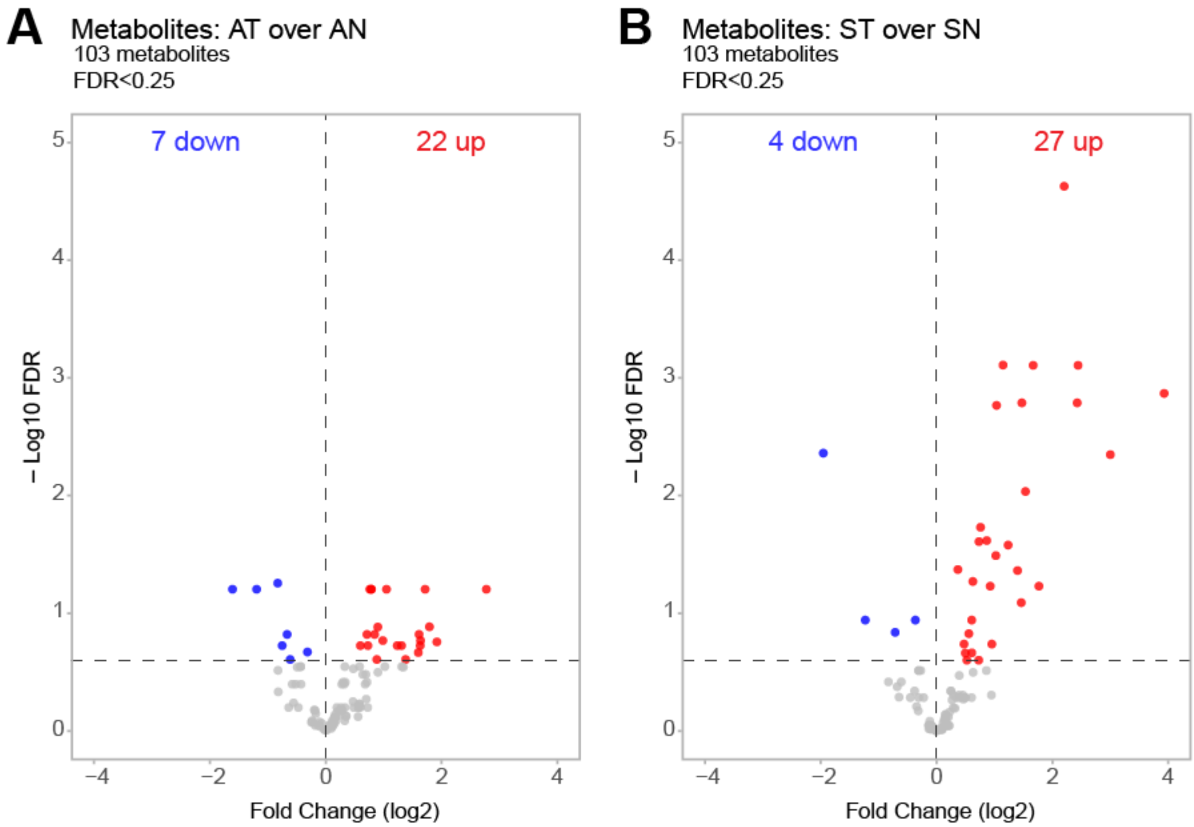

Supp Figure 3

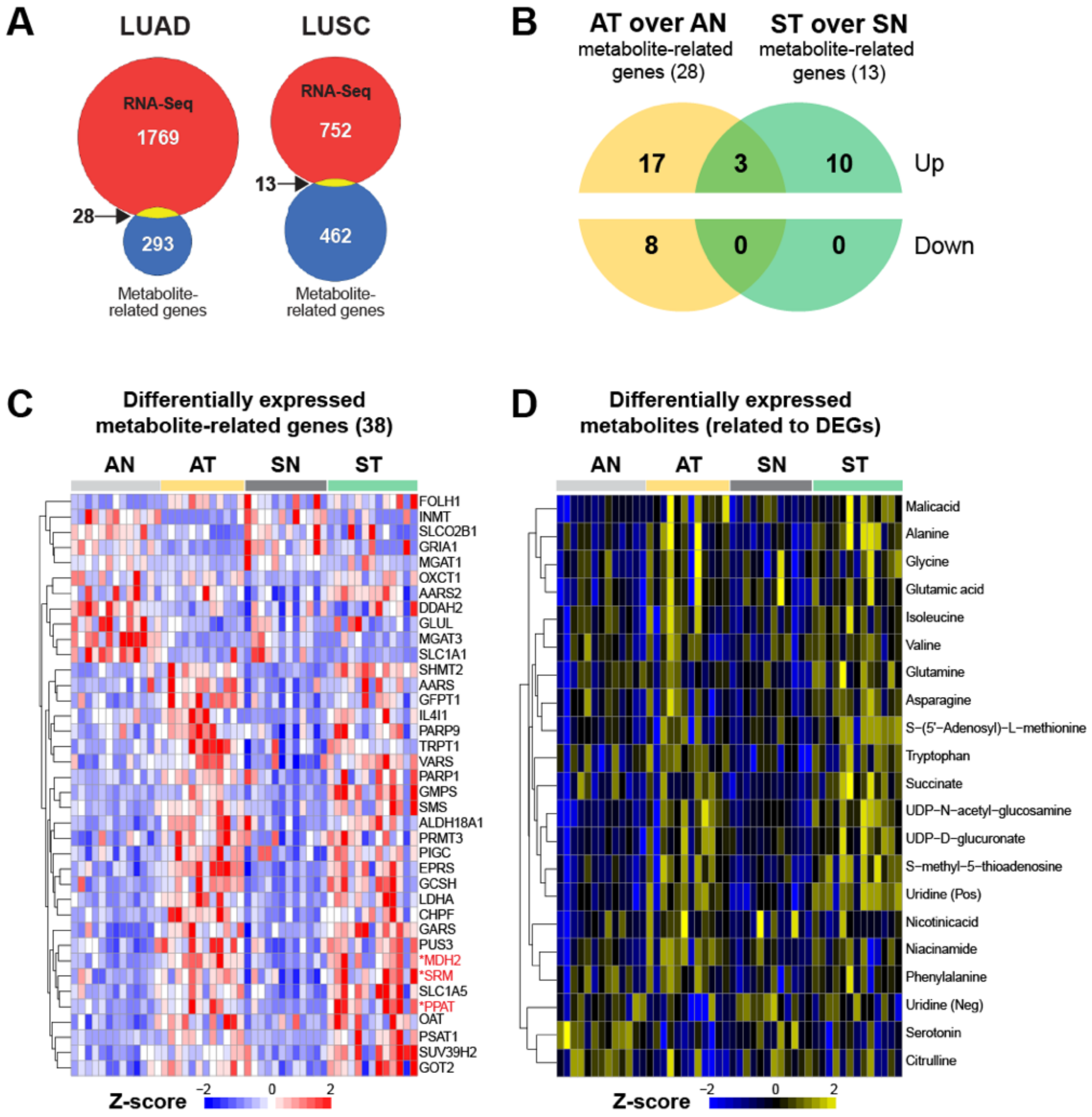

Supp Figure 4

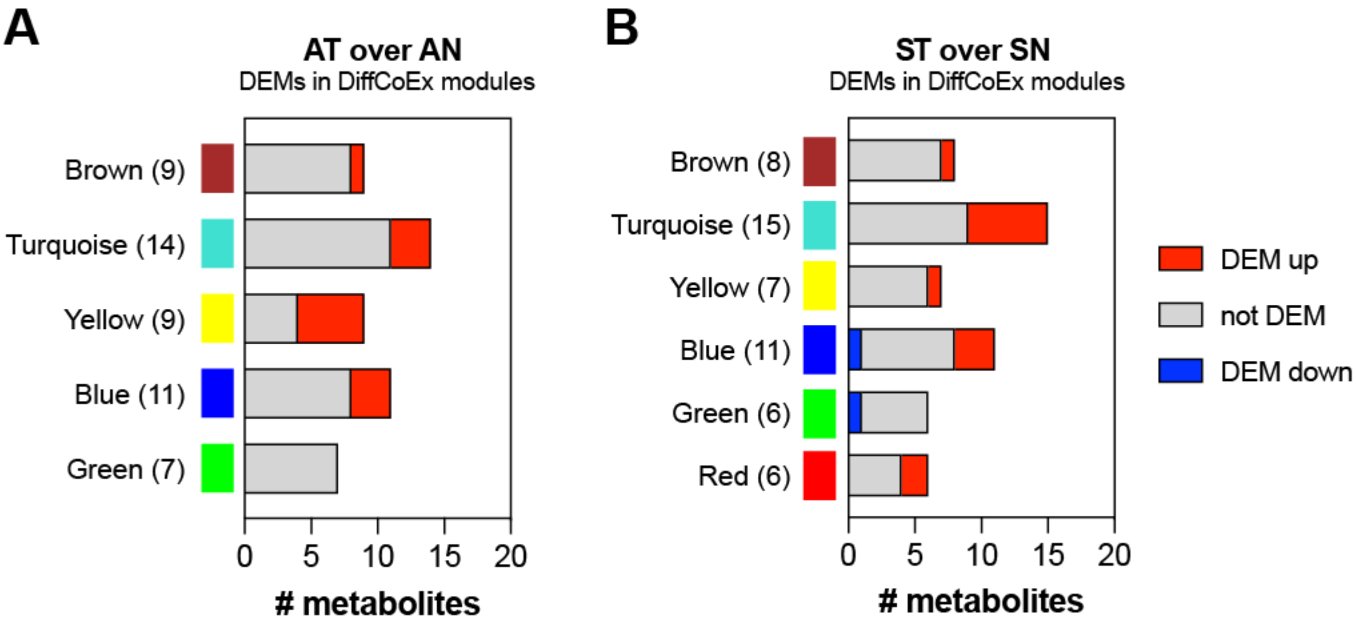

Supp Figure 5

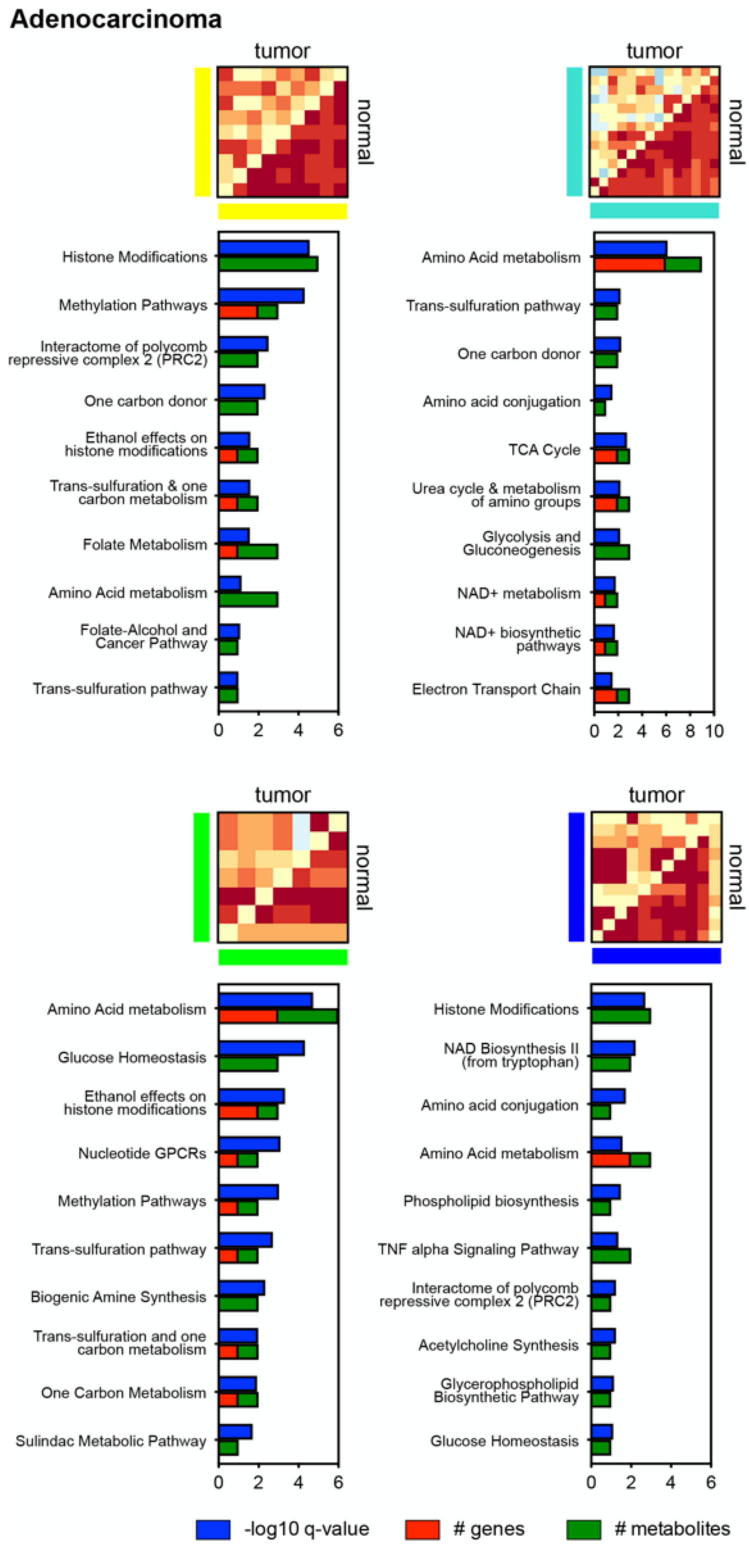

Supp Figure 6

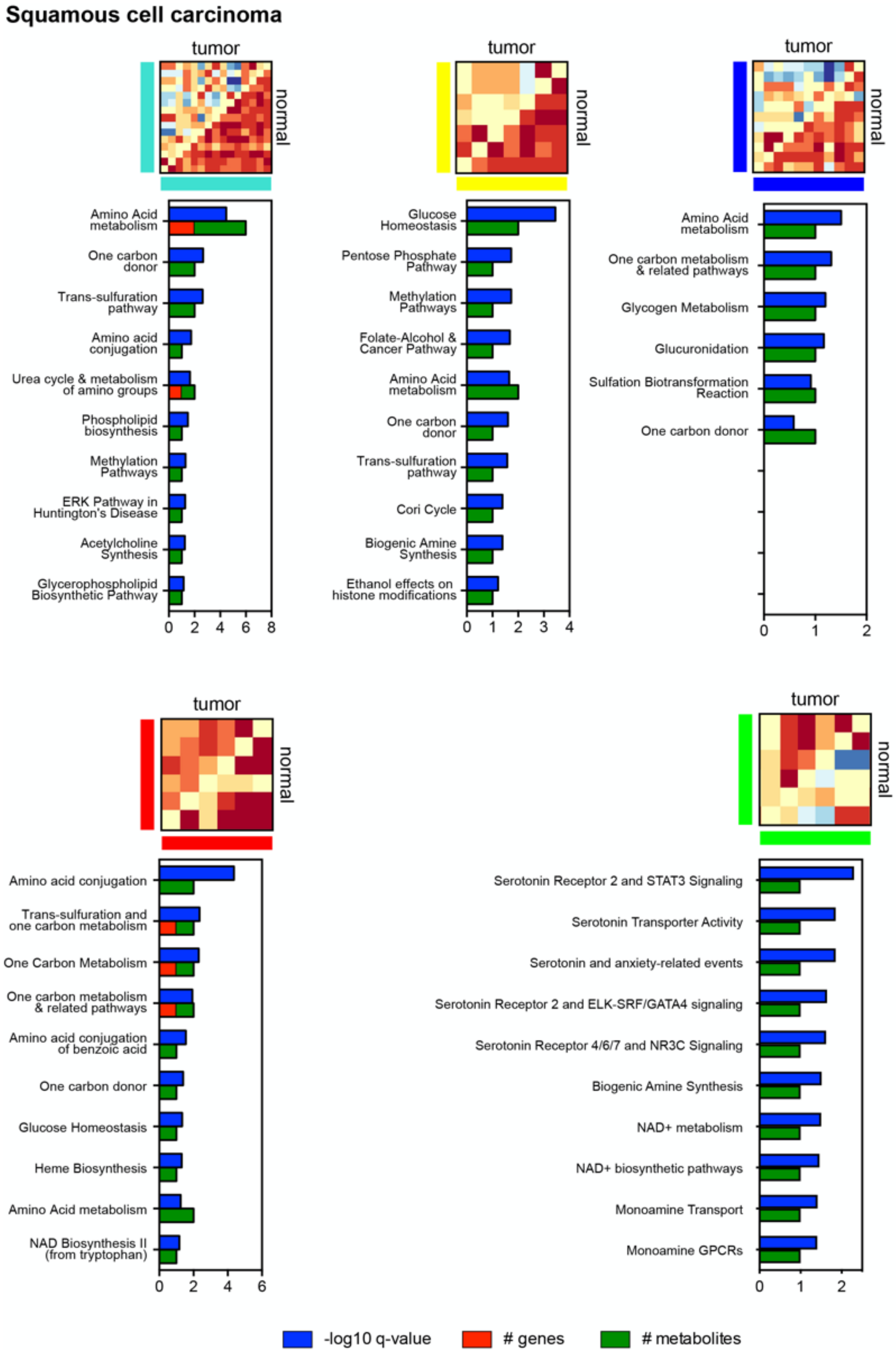

Supp Figure 7

	1	2	3	4	5	6	7	8	9	10	11	12	
A	ALDH18A1	PIGC	UBE2T	MYBL2	TPX2	PLK1	ALDH18A1	PIGC	UBE2T	MYBL2	TPX2	PLK1	A
B	CHPF	PPAT	EXO1	HIST1H3B	ESCO2	NEK2	CHPF	PPAT	EXO1	HIST1H3B	ESCO2	NEK2	B
C	EPRS	PSAT1	CENPA	CHODL	ASF1B	AURKB	EPRS	PSAT1	CENPA	CHODL	ASF1B	AURKB	C
D	FOLH1	SMS	UBE2C	GPR87	HIST1H2AH	STEAP1	FOLH1	SMS	UBE2C	GPR87	HIST1H2AH	STEAP1	D
E	GFPT1	TCN1	CCNB1	ANLN	KIF2C	ACTB	GFPT1	TCN1	CCNB1	ANLN	KIF2C	ACTB	E
F	LDHA	ANXA10	DEPDC1B	FGF11	HIST1H3B	GAPDH	LDHA	ANXA10	DEPDC1B	FGF11	HIST1H3B	GAPDH	F
G	MDH2	ADAMDEC1	CYP19A1	RRM2	PSAT1	RQ1	MDH2	ADAMDEC1	CYP19A1	RRM2	PSAT1	RQ2	G
H	OAT	EPHX4	SPDEF	HIST1H2BO	PYCR1	gDNA	OAT	EPHX4	SPDEF	HIST1H2BO	PYCR1	gDNA	H
	1	2	3	4	5	6	7	8	9	10	11	12	

Supp Figure 8

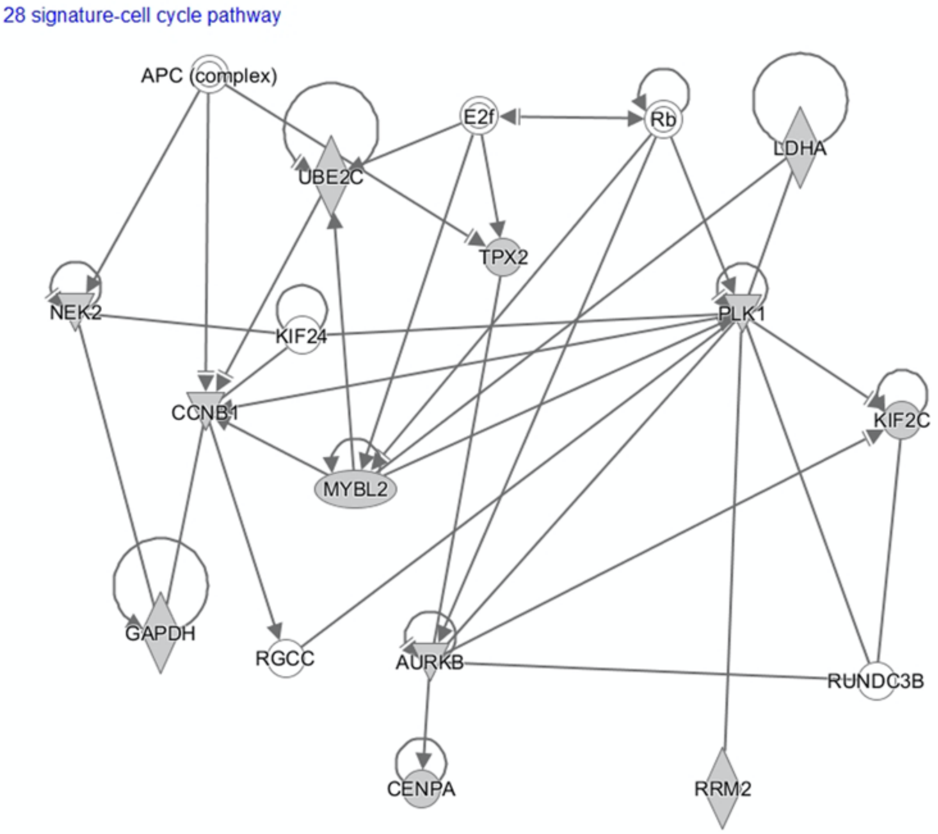

Supplement: Supplementary file 1 — Figure S1–S8 [file CAM4-12-584-s001.zip › cam44933-sup-0001-FiguresS1-S8.pdf]
