# Supplementary material for: Integrative metabolomics and transcriptomics analysis reveals novel therapeutic vulnerabilities in lung cancer
Source: Cancer Med. 2022 Jun 8;12(1):584–96. doi: 10.1002/cam4.4933 (PMC9844651; doi:10.1002/cam4.4933)
Supplement: Supplementary file 1 — Figure S1–S8 [file CAM4-12-584-s001.zip › CAM4_4933_CancerMed_SuppFig_legends_4.22.22.docx]

**Supplemental Figure Legends:**

**Figure S1: Overlap and heatmap of combined LUAD and LUSC signature genes.** A. Venn diagram showing overlap of differentially expressed genes identified for LUAD (AT over AN) and LUSC (ST over SN). B. Heatmap showing scaled expression of differentially expressed genes in LUAD and LUSC (FDR<0.05, fold change exceeding 1.25×). Each subset from the Venn diagram in panel A is represented in the heatmap, indicated on the right.

**Figure S2: Differentially expressed metabolites (DEMs).** Volcano plots for LUAD (A) or LUSC (B), showing log2 fold change on the x-axis and significance (–log10 FDR) on the y-axis. Metabolites showing a significant difference at FDR<0.25 are colored red (up) or blue (down), with non-significant metabolites colored gray. A. For metabolites increased in LUAD the fold change range is 1.51-6.83x, whereas for the decreased metabolites is 1.24-3.05x. B. For metabolites increased in LUSC the fold change range is 1.29-15.24x, whereas for the decreased metabolites is 1.29-3.88x.

**Figure S3: Overlap and heatmap of differentially expressed metabolite-related genes and associated DEMs.** A. Venn diagrams from main Figure 2, showing overlap of differentially expressed genes identified by RNA-seq with differentially expressed metabolite-related genes for LUAD or LUSC, added for clarity. B. Venn diagram of 28 LUAD and 13 LUSC metabolite associated differential genes identified in panel A, showing that 3 cancer increased genes are common between LUAD and LUSC. C. Heatmap showing scaled expression of differentially expressed metabolite-related genes in LUAD and LUSC (FDR<0.05, fold change exceeding 1.25×). Common DEGs between LUAD and LUSC are indicated with an asterisk and colored red. D. Heatmap showing scaled expression of differentially expressed metabolites in LUAD and LUSC, related to genes shown in panel C (FDR<0.25).

**Figure S4: Overlap between DiffCoEx modules and differentially expressed metabolites (DEMs).** Bar plots show differentially expressed metabolites (FDR<0.25) from LUAD (A) or LUSC (B) that were identified in each module from DiffCoEx analysis. Metabolites were either up-regulated (red), down-regulated (blue), or not differentially expressed (gray).

**Figure S5: Pathway enrichment in LUAD using DiffCoEx identified genes and metabolite- related genes.** Top ten enriched pathways in LUAD using metabolites from the yellow, turquoise, green, and blue module DiffCoEx modules (graphed as –log10 p- value), with the number of metabolites or metabolite-related genes contributing to pathway enrichment. Significance was determined by hypergeometic distribution.

**Figure S5: Pathway enrichment in LUSC using DiffCoEx identified genes and metabolite-related genes.** Top ten enriched pathways in LUSC using metabolites from the turquoise, yellow, blue, red, and green DiffCoEx modules (graphed as –log10 p- value), with the number of metabolites or metabolite-related genes contributing to pathway enrichment. Significance was determined by hypergeometic distribution. For the blue module, only 6 pathways in total were enriched.

**Figure S7**: **Custom RT-PCR** **plate map of 28 metabolite gene signature and cell cycle genes.** A plate map shows 42 genes in duplicate, in addition to *ACTIN* and *GAPDH* as reference controls, used to perform RT-PCR to assess their expression.

**Figure S8**: **LDHA interacts with cell cycle genes**. We used IPA software (IPA- Qiagen) to identify genes that interact with LDHA. LDHA, one of the upregulated metabolite-related genes that had a significant association with survival in LUAD, interacts with a network of key cell cycle genes such as PLK1, AURKB, CCNB1, CENPA, and KIF2C in addition to other genes.
